# Principles for an Implementation of a Complete CT Reconstruction Tool Chain for Arbitrary Sized Data Sets and Its GPU Optimization

**DOI:** 10.3390/jimaging8010012

**Published:** 2022-01-15

**Authors:** Jürgen Hofmann, Alexander Flisch, Robert Zboray

**Affiliations:** Center for X-ray Analytics, Empa, Swiss Federal Laboratories for Materials Science and Technology, Überlandstrasse 129, 8600 Dübendorf, Switzerland; alexander.flisch@empa.ch (A.F.); robert.zboray@empa.ch (R.Z.)

**Keywords:** computed tomography, CT reconstruction software, GPU-based reconstruction, bad center of rotation correction, data splitting techniques

## Abstract

This article describes the implementation of an efficient and fast in-house computed tomography (CT) reconstruction framework. The implementation principles of this cone-beam CT reconstruction tool chain are described here. The article mainly covers the core part of CT reconstruction, the filtered backprojection and its speed up on GPU hardware. Methods and implementations of tools for artifact reduction such as ring artifacts, beam hardening, algorithms for the center of rotation determination and tilted rotation axis correction are presented. The framework allows the reconstruction of CT images of arbitrary data size. Strategies on data splitting and GPU kernel optimization techniques applied for the backprojection process are illustrated by a few examples.

## 1. Introduction

At Empa’s Center for X-ray Analytics, different types of Computed Tomography (CT) scanners are in use. They encompass a cone-beam CT scanner with sub-micrometer resolution, micro-CT scanners in the higher energy regime (up to 300 keV), X-ray phase contrast instruments and a high-energy CT system (up to 6 MeV) using a linear accelerator as an X-ray source. In this article, tools for the complete CT reconstruction chain are presented. They have been in use for many years for in-house CT reconstruction. The basic principles and the implementation of artefact reduction methods will be discussed. Furthermore, we will give insights into implementation and optimization strategies. We started the development of this in-house software for CT reconstruction to satisfy needs that could not be covered by commercial software. Since the CT data sets were growing over time with the increase in detector resolution from approximately 512 × 512 pixels to 4096 × 4096 pixels today by more than an order of magnitude, an according speed up in reconstruction time was also becoming mandatory. Therefore, we implemented the backprojection, the most time-consuming component, on a graphics processing unit (GPU) using NVIDIA’s CUDA toolkit [1]. This allows fast—a few minutes—backprojection for large-volume data sets (≈2048${}^{3}$ voxels). In the last 10 years, we continuously integrate new modules capable of handling artefact reduction and geometry correction. Graphical user interfaces for all modules easily allow parameter configuration and the execution of the applications. Nevertheless, it is also possible to run the filtered backprojection headless, which allows batch processing. The reconstruction of phase-contrast CT will be described elsewhere. Nowadays several free or open source reconstruction tool kits are existing. TIGRE [2], ASTRA [3], RTK [4] and MuhRec [5] are prominent examples. TIGRE, ASTRA and RTK providing iterative solvers, which are capable of handling reconstruction problems that cannot be solved by filtered backprojection. These are, e.g., limited angle CT or the integration of physical models in the reconstruction procedure. Reviews for the different methods are presented in [6,7]. Early examples for CT reconstruction implementation on the GPU are those from Fang Xu et al. [8] using GPU shaders and Holger Scherl et al. [9] using CUDA textures. However, the usability was limited due to the small GPU memory size (NVIDIA GeForce 8800 GT 512 MB). The majority of the tool kits today are using GPUs for process acceleration. Specific for our implementation is the automated determination of the center of rotation and the rotation axis tilt. These features are essential in practice for the reconstruction, especially with high magnification measurements using nano- and micro-cone-beam CT systems. Our implementation running under Windows is available as open source at: https://github.com/JueHo/CT-Recon, accessed on 15 December 2021.

## 2. Methods: Overview of the Reconstruction Framework

**Figure 1:**
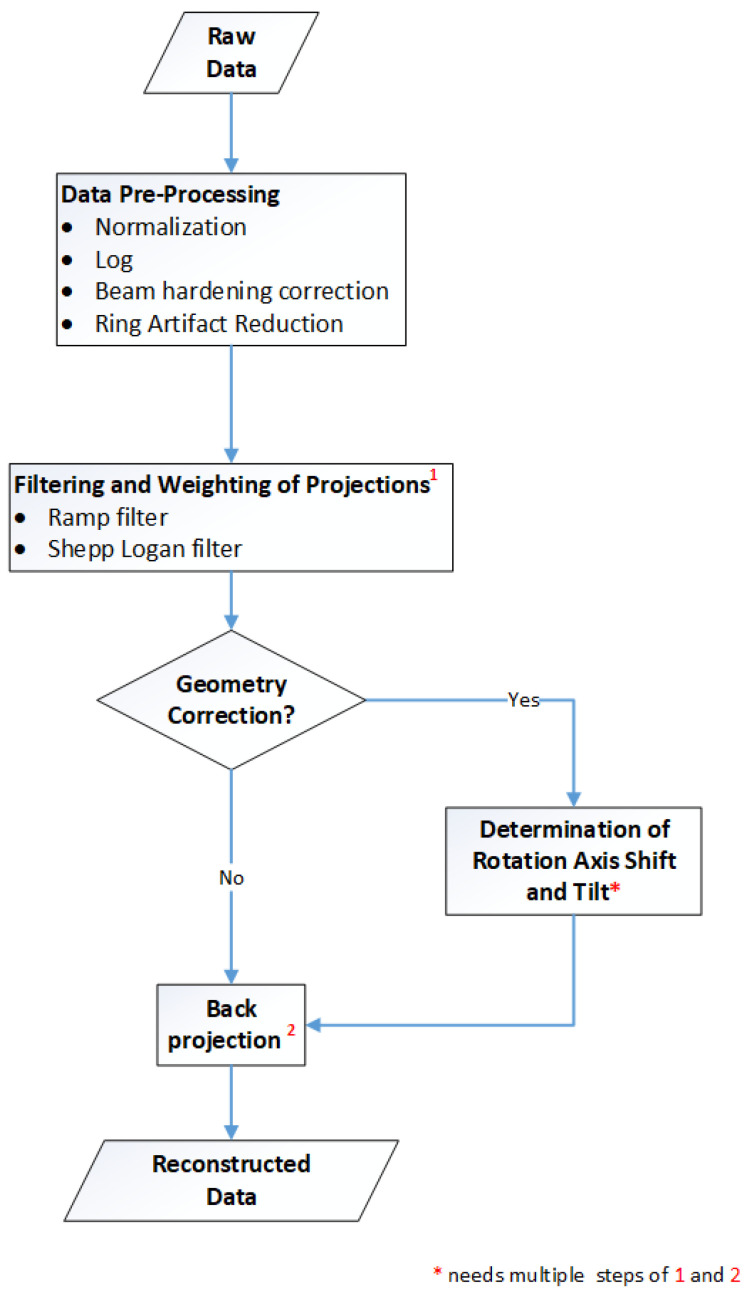
Overview: Workflow of CT reconstruction.

Figure 1 gives an overview of the reconstruction framework: the modules, the processes and the data flow. The next chapters focus on the method and implementation description of the two main parts of the CT reconstruction: the pre-processing of the raw data and the filtered backprojection. These include ring artefact reduction methods, automatized estimation of the rotation axis offset, tilted axis correction and a simple beam hardening correction. The basic principles for the Lak-Ram and Shepp-Logan filter design are explained. For the backprojection step we present details on the implementation of the Feldkamp–David–Kress algorithm (FDK) [10] on GPU. Furthermore a data-splitting algorithm is introduced, which enables the reconstruction of arbitrarily sized volumes.

### 2.1. Pre-Processing

Pre-processing includes several steps before executing the actual CT reconstruction with the filtered backprojection. It involves mandatory processing steps such as normalization and taking the logarithm of the normalized projection images. Others methods such as those for ring artifact suppression and beam hardening correction are optional. In the subsequent paragraphs, we explain all methods in detail.

#### 2.1.1. Normalization and Beam Hardening Correction

Normalization:

Raw projection images have to be corrected by a reference image taken without the object, the flat-field image and a dark-field image acquired without X-ray radiation. The dark- and flat-field correction as shown in Equation (1) is normally performed during the data acquisition. Scanner systems typically provide internally an up-scaling to the original data range or a fluence correction. Therefore, we introduce the term fluence (integrated counts on the detector over the exposure time) $C_{fl}$ in Equation (1), which should make our further statements clearer:

(1) $$I_{proj}^{\varphi_{i}}\left(x,y\right)=\frac{I_{raw}^{\varphi_{i}}\left(x,y\right)-I_{dark}\left(x,y\right)}{I_{flat_{raw}}\left(x,y\right)-I_{dark}\left(x,y\right)}C_{fl}$$

with projection at angle $\varphi_{i}\in \left[0,2\pi \right]$.

We distinguish between two cases of up-scaling ($C_{fl}$) performed during data acquisition:

1. The data range after the normalization will be up-scaled to the level of the raw projections, e.g., in 16-bit representation, before normalization ($C_{fl}$ Equation (2)):
  (2) $$C_{fl}=mean\left(I_{flat_{raw}}\left(x,y\right)\right)-I_{dark}\left(x,y\right));$$
2. An additional measurement device will gather the fluence of each projection ($C_{fl}$ Equation (3)):
  (3) $$C_{fl}=I_{fl}^{\varphi_{i}}-mean\left(I_{dark}\left(x,y\right)\right).$$

**Figure 2:**
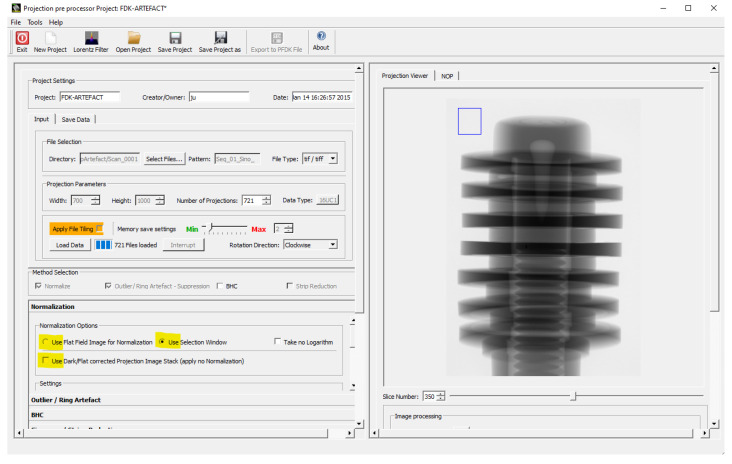
ROI selection used for fluence up-scaling.

In our implementation, we have three options to consider the different cases (see Figure 2, options yellow marked):

1. No correction. The projections are already fluence corrected.
2. The projections are only up-scaled, and it is possible to select a background ROI for a post-fluence correction. Then, we use Equation (4) for the correction.
  For that, a region of interest (ROI) of the background is taken for every projection at the same position. It is used to perform a fluence correction of the current projection by the mean value of the pixels values within the ROI. The selection of the ROI is performed interactively, as illustrated in Figure 2. The ROI should not interfere with the object, which needs to be verified by stepping through all projections:
  (4) $$I_{corr}^{\varphi_{i}}\left(x,y\right)=\frac{I_{proj}^{\varphi_{i}}\left(x,y\right)}{mean(win^{\varphi_{i}})}$$
3. The projections are only up-scaled, but it is not possible to select a background ROI and perform a fluence correction. This might be because, e.g., projections are completely filled by the object. Then, we use Equation (5) to re-scale the projections to make the gray values comparable to those corrected by Equation (4):
  (5) $$I_{corr}^{\varphi_{i}}\left(x,y\right)=\frac{I_{proj}^{\varphi_{i}}\left(x,y\right)}{mean(I_{flat_{raw}}\left(x,y\right)-I_{dark}\left(x,y\right))}$$
  Obviously for the in-house scanner where all the images (projection, flat and dark) are available, the user can provide those to our package and choose from the above option and use the respective equations. Our package flexibly supports all these input image options.

**Figure 3:**
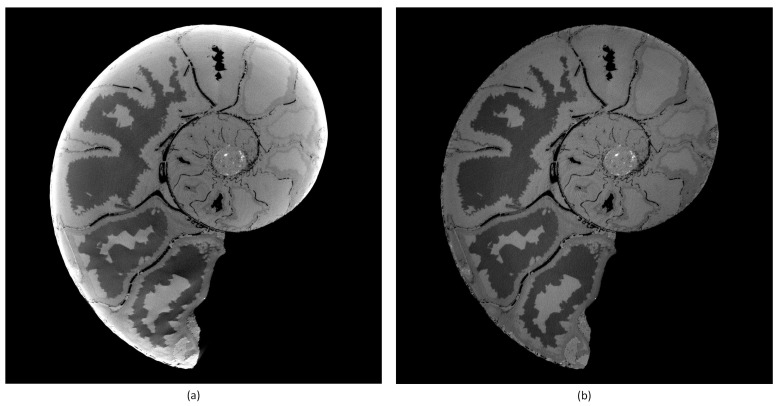
Tomogram of an ammonit. Left (**a**) without BHC. Right (**b**) with BHC. Parameters: *a* = 1.0, *b* = 1.0, *c* = 3.0.

Beam hardening correction (BHC):

Bremsstrahlung-based X-ray sources typically used in laboratory CT systems have a polychromatic energy spectrum. Beam hardening occurs when an X-ray beam consisting of polychromatic energies passes through an object, and the sample material attenuates more pronounced lower energy photons. The mean value in the X-ray spectrum reaching the detector is shifted to higher energy. The result is a non-linearity of the attenuation versus the material thickness. In Figure 3, the artefact caused by beam hardening is visible in higher gray values in the outer region and lower gray values in the center of the object’s tomogram, as the lower energy photons preferentially become attenuated over longer path lengths. We chose a polynomial approach [11,12] for the correction of beam hardening, which is fast and delivers good results in practice, especially applied to cupping artifacts. The function-based correction method—e.g., with polynomials—linearizes the attenuation values in the projections. Equation (6) shows the expression used for the correction. For the correction of severe beam hardening artifacts such as photon starvation, an iterative correction algorithm [13] is often more suitable:

(6) $$f\left(x\right)=a\cdot x+b\cdot x^{c}\;\;x:\;\mathrm{image}\;\mathrm{gray}\;\mathrm{value}\;\mathrm{and}\;a,\;b,\;c:\;\mathrm{empirical}\;\mathrm{coefficients}$$

The parameters used for BHC are based on experience. Typically, values for *c*, the most sensitive parameter, are in the range of [2.0, 3.0].

#### 2.1.2. Ring Artifact Reduction

**Figure 4:**
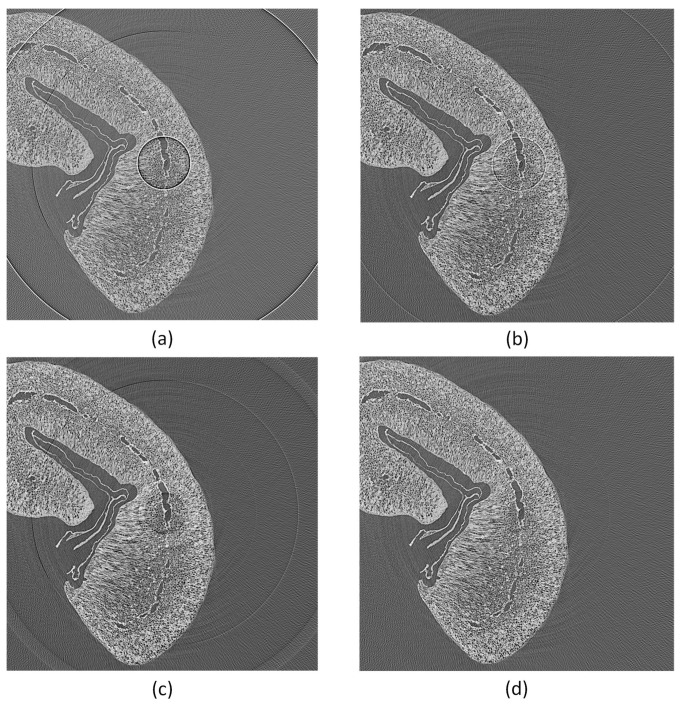
Tomogram of a coffee bean. Applying outlier and ring artifact reduction methods: (**a**) without any corrections (two to three bad pixels next to each other cause severe ring artifacts); (**b**) with MF method only; (**c**) with DLR correction only; (**d**) with MF and DLR method together.

Typical ring artifacts can be observed in the CT slice in Figure 4a. We differentiate between two main types of ring artifact sources. The first is caused by malfunctioned detector pixels creating severe artifacts. The second is caused by the nonuniform sensitivity of the detector pixels, which is less severe, but still induces significant ring artifacts in the reconstructed images. We implemented two methods to mitigate ring artifacts.

The first method is based on median filtering (MF), which reduces coarse ring artifacts induced by defect pixel values. As input for this method, we define a threshold value $\sigma_{th}=n*\sigma$ for the gray value outliers as a multiple of the standard deviation. In the projection stack, structures and edges vary from image to image contrary to the defect pixels, which stay on the same coordinates. Therefore, we sum up all projection images pixel-wise into an image $I_{su}$. This reduces the influence of actual edges in $I_{su}$. To detect the outliers, we build the difference image $I_{diff}$ of $I_{su}$ and the median blurred of $I_{su}$. We build a z-score [14] image from $I_{diff}$ and create an outlier image mask $I_{mask}$, as described in Algorithm 1. The mask *I_mask_* is used in a median filtering procedure for the X-ray projections, as shown in Algorithm 2. This MF method mitigates coarse ring artifacts caused by defect pixel values and defect detector lines (See changes in images in Figure 4a,b). The algorithm utilizes functions from the Open Source library OpenCV [15] for its implementation.
**Algorithm 1:** Find outliers in X-ray image stack.**Input**: Image stack of X-ray projection, Threshold value σ${}_{t}$${}_{h}$ of outliers, window mask win(w = width, h = height)**Output**: Image mask $I_{mask}$ with outlier pixels// Average pixel wise over X-ray projection stack $P_{i}$$\tilde{P}$ = $\frac{1}{n}\sum_{i=1}^{n}P_{i}$MI = MedianBlur($\tilde{P}$, win(w, h))// Pixel wise absolute difference Image DI between//averaged projections and median blur image// => enhance outliersDI = |$\tilde{P}$− MI|// calculate z-score of DIs = stdev(DI) // standard deviationm = mean(DI) // mean valueI_z-score_ = $\frac{\mathrm{DI}-m}{s}$// z-score of image$I_{mask}$ = threshold(I_z-score_, σ${}_{t}$${}_{h}$, BINARY_MODE)**return***I_mask_*

**Algorithm 2:** Median filtering of defect pixels in X-ray projections.

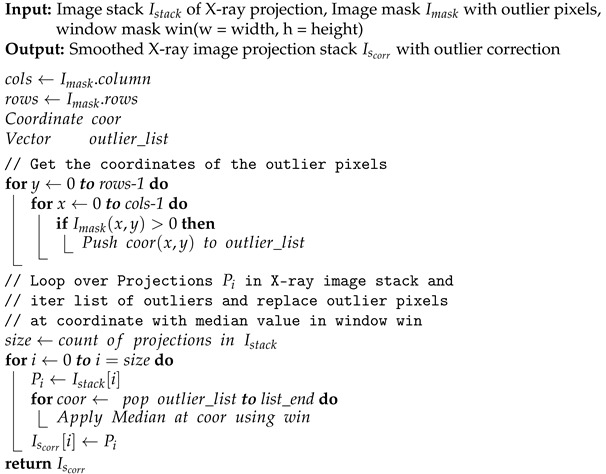



The second method is proposed by Y. Kim et al. [16] and is based on ring artifact correction using detector line ratios (DLR). The objective of this method is the build-up of a sensitivity correction matrix (SCM) from the projection image stack. The SCM is used to correct the pixel values of all projections P(φ) according to the determined detector sensitivity. The implementation in our pre-processing application is following closely the description in the paper.

The best results in ring artifact reduction can be accomplished by combining both methods, first applying the outlier correction method and afterwards the process projections detector sensitivity equalization method. An example is shown in Figure 4. Only applying the MF method (Figure 4b) leads to a reduction of ring artifacts but still leaves significant rings visible. The DLR method (Figure 4c) alone is not capable of handling defect pixels well and introduces additional artifacts. Both methods applied together (Figure 4d) show good results in ring artifact suppression.

### 2.2. Weighting and Filtering of Projections

**Figure 5:**
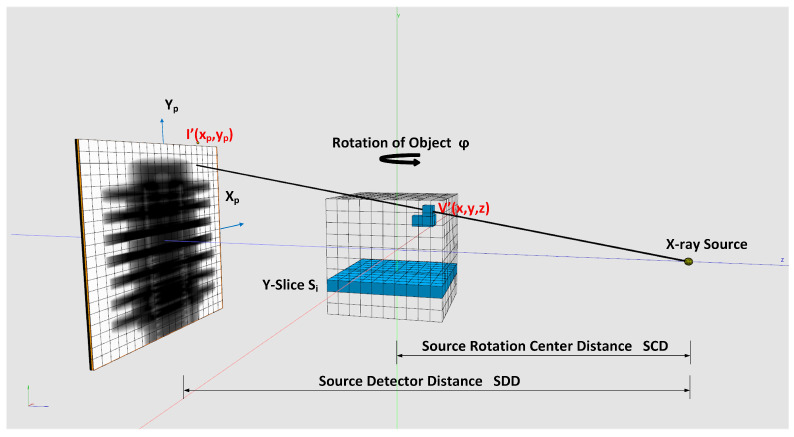
Voxel-driven backprojection.

Weighting: For cone-beam CT scanners, it is necessary to weight the pixel gray values in the projection images according to their geometry. We use the geometry notation of Figure 5 for the weighting shown in Equation (7). When the rotation axis is not centered, the pixel positions in $X_{p}$ must be shifted by the offset value determined in Section 2.4.1:

(7) $$\begin{array}{c} W\left(X_{p},Y_{p}\right)=\frac{SDD}{\sqrt{X_{p}^{2}+Y_{p}^{2}+SDD^{2}}} \end{array}$$

Weighting of pixel values in a projection.

Filtering: The ramp (Ram-Lak) filter is an integral part of the filtered backprojection and is therefore mandatory. It has its mathematical origin in the Jacobian determinant for the Polar to Cartesian transformation, which has to be applied for the derivation of the backprojection algorithm [10]. The discrete Ram-Lak (Equation (8)) and Shepp and Logan filter (Equation (9)) are integrated in the reconstruction framework. Δs represents the sampling interval. The derivation of the discretized Ram-Lak and Shepp-Logan filters can be found in [10,17]. The discrete filters are implemented in the spatial domain to avoid constant offset (DC) errors. This is because the digital measurement of the projections is band limited due to discrete data sampling. A non-discrete filter would cause a DC offset error, which cannot be completely eliminated by zero padding [10]. The convolution of the filter kernel with the projections are processed in Fourier space due to performance reasons. Filtering is applied row wise. Filter and projections are zero padded. The padding length is at least $N_{f}+N_{p}-1$, where $N_{f}$ is the filter length and $N_{p}$ is the projection length. This avoids interference errors are caused by acyclic convolution.

(8) $$H_{RL}\left(n\Delta s\right)=\left\{\begin{array}{cc} \frac{1}{4\Delta s^{2}} & \mathrm{if}\;n\;=\;0.\\ 0 & \mathrm{if}\;n\;\mathrm{is}\;\mathrm{even}\;(n\neq 0).\\ -\frac{1}{{\left(n\pi \Delta s\right)}^{2}} & \mathrm{if}\;n\;\mathrm{is}\;\mathrm{odd} \end{array}\right.$$

Discrete Ramachandran and Lakshminarayanan filter kernel in spatial domain.

(9) $$H_{SL}\left(n\Delta s\right)=-\frac{2}{{\left(n\pi \Delta s\right)}^{2}}\frac{1}{4n^{2}-1}$$

Discrete Shepp and Logan filter kernel in spatial domain.

### 2.3. Backprojection

In our framework, we implemented the voxel-driven method for backprojection. A ray is going from the X-ray source through the center of the voxel under reconstruction and is intersecting the projection plane afterwards. Code Listing A1 shows the implementation. The interpolated gray value at the intersection point is taken to sum up the voxel value. To obtain sub-pixel accuracy, bi-linear interpolation of the gray values in the neighborhood of the intersection point is performed. We provide two implementations for the bi-linear interpolation. The first is a fast hardware accelerated method that uses CUDA 2D texture fetching function tex2DLayered(). This method uses 9-bit fixed point format with 8 bits of fractional value [18], which givest the best accuracy for values near 1.0. For better accuracy in case of values far from 1.0, we implemented an interpolation based on a 32 bit floating point function—tex2DLayeredHighPrec()—running in a CUDA kernel function fdk_kernel_3DW_HA(). For details on implementation, see source code Listing A2. Using function tex2DLayeredHighPrec() decreases backprojection speed approximately by a factor of two. The backprojection instruction is shown in Equation (10). Voxels are reconstructed slice-wise $S_{i}$ as seen in Figure 5. The input data for the reconstruction are the weighted and filtered projections as described in Section 2.2.

(10) $$V^{\prime }\left(x_{i},y_{i},z_{i}\right)=\sum_{\varphi =0}^{2\pi }\frac{SCD^{2}}{{\left(SCD-z_{i}\left(\varphi \right)\right)}^{2}}I_{\varphi }^{{}^{\prime }}\left(X_{p},Y_{p}\right)$$

$z_{i}\left(\varphi \right)$: Projection on *z* of voxel $V_{i}^{{}^{\prime }}$ in volume coordinates and an angle φ.

$I_{\varphi }^{{}^{\prime }}\left(X_{p},Y_{p}\right)$: Weighted and interpolated pixel value of a projection at the angle φ.

Discrete backprojection (see Figure 5 and details on implementation in Listing A3).

**Figure 6:**
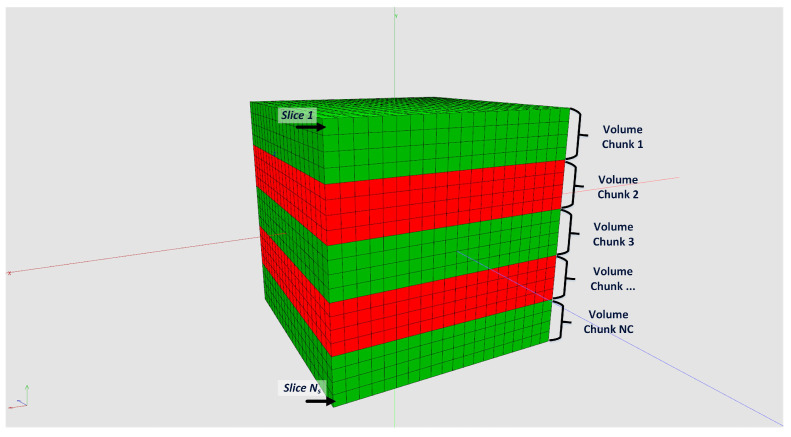
Splitting reconstruction volume into chunks, which are small enough to fit into CPU memory.

**Figure 7:**
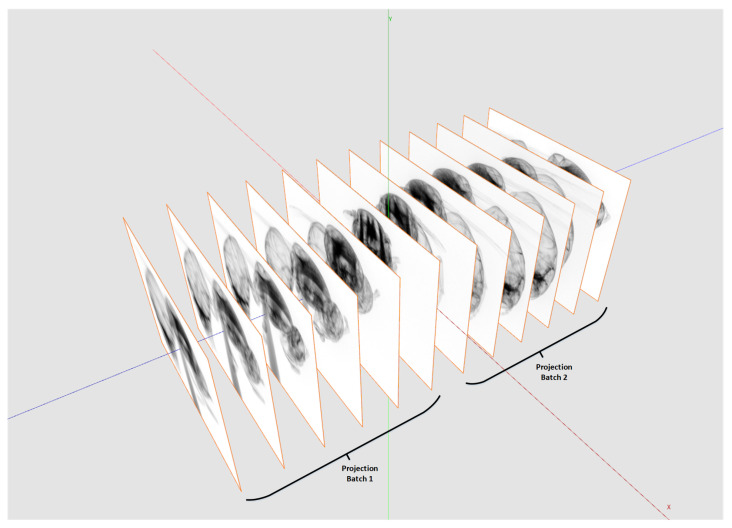
Depending on the available GPU memory and the maximal GPU texture size, the projection stack used for the reconstruction needs to be split.

One main feature of our framework is the capability to reconstruct tomograms from arbitrarily sized raw data sets. Neither the input data nor the reconstructed volume have to fit into CPU or GPU memory. We achieve this by splitting input data and reconstruction volume size according the available hardware resources. For that, the actual free CPU and GPU memory must be estimated and compared with the required resources. Figure 6 shows how to split the reconstruction volume so that every chunk fits into the memory. This partitioning is possible because every voxel can be reconstructed independently from each other. For the backprojection, the projection data are uploaded to 2D layered textures on the GPU. However, since the GPU memory compared to the CPU memory is typically much more limited, the complete projection dataset usually does not fit into the GPU memory. The backprojection runs over all projections from 0${}^{\circ }$ to 360${}^{\circ }$. Therefore, the complete backprojection process of a voxel can be divided into a sequence of sub-backprojections using batches of projections which are fitting into the GPU memory (see Figure 7). The Algorithm 3 shows the workflow of the sub-divided reconstruction process in more detail.

### 2.4. Geometry Correction

Because data acquisition with CT scanners always contains errors regarding the geometry, post corrections have to be applied for accurate reconstruction results. In our application, we integrated the correction of the horizontal offset of the rotation axis and the axis tilt. The presented algorithm can also be applied for other geometry corrections, e.g., a slanted detector.    
**Algorithm 3:** Backprojection of arbitrary data size. Reconstruction workflow for the partitioning of input data not fitting in CPU or GPU memory.
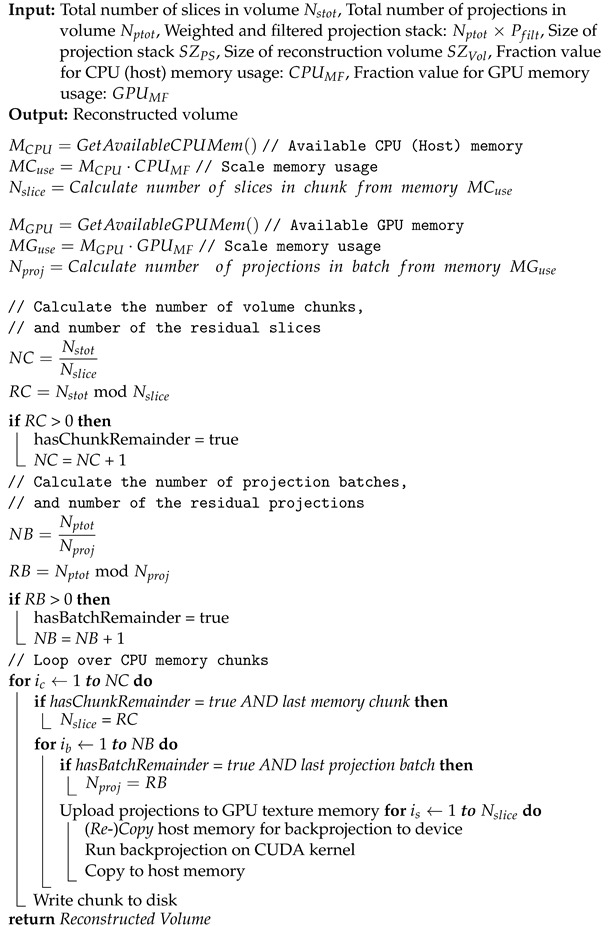


#### 2.4.1. Out of Center Correction for the Rotation Axis

**Figure 8:**
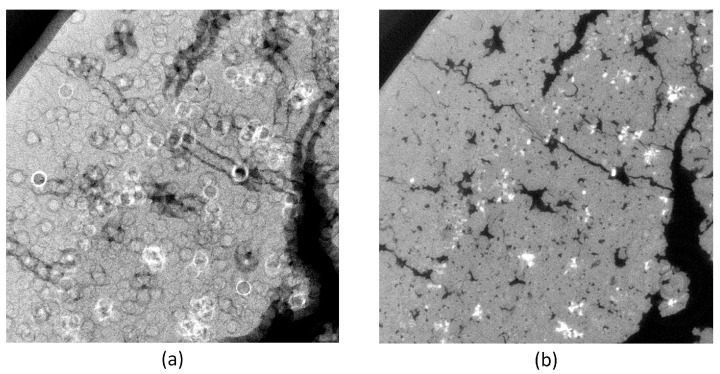
Detailed view of a tomogram (Cheese with salt particles): (**a**) Left image without rotation axis correction; (**b**) right image rotation axis corrected with −15.2 pixels horizontal shift.

A rotation axis out of center leads to the artifacts seen in Figure 8a. The determination of the horizontal rotation axis shift is performed on reconstructed tomograms. For a free selectable slice in the tomogram stack (best near the central slice, because of the absence of cone-beam artifacts) a sequence of tomograms with increasing rotation axis offsets is reconstructed. The method to find the correct rotation axis offset is based on the autofocus principle known from cameras and is adapted for X-ray CT [19]. By selecting the image with the sharpest edges, we obtain the correct axis offset. As we see in Figure 8a, an image without offset correction is blurred. The evaluation of the gray values of the gradient images is used to determine a qualifying sharpness score. For a successful application of the method in practice, some additional image processing steps are necessary, e.g., denoising. For implementation details, see the description of Algorithms 4 and 5. It is crucial to select a slice with structures and edges. In images with almost no structures or edges, the algorithm may fail, similar to autofocus cameras.
**Algorithm 4:**T_score_ Determine sharpness score.**Input**: Tomo slice image S, width of tomogram w, margin factor m < 1**Output**: Sharpness score of tomo slice $T_{s}$Calculate length L = m · w · 1/$\sqrt{2}$ for processing windowCopy centred processing sub window image I_sw_ with size L × L Edge preserving smoothing of image $I_{s}$ using Bilateral FilterCalculate derivative image I_sob_ using Sobel Filter//Determine sharpness score over all pixelsT_score_ = Σ I_sob_· I_sob_// Make Pixel-Values ≥ 0 (Isob${}^{2}$) and accumulate**return***T_score_*

**Algorithm 5:**$X_{m}$Find shift of rotation axis (uses Algorithm 4 “Determine sharpness score”).

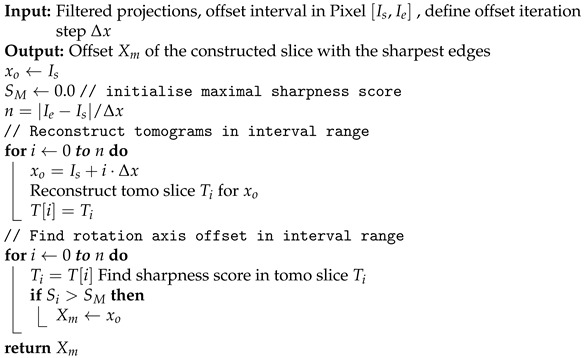



#### 2.4.2. Rotation Axis Tilt Correction

If the rotation axis offset is not constant along all slices in the tomogram stack and we have a linear dependency of the offset and the position in the tomogram stack $z_{i}$, we speak of a tilted rotation axis. We can compensate the tilt by considering the functional dependency of the tomogram stack position and the axis offset in the slice. For that, we have to find the parameters of the equation f(x):

(11) f(x)=a+m·x

with

$$f\left(x\right)=\left(\begin{array}{c} f(x_{1})\\ f(x_{2})\\ f(x_{3})\\ \vdots \\ f(x_{m}) \end{array}\right),\;X=\left(\begin{array}{cc} 1 & x_{1}\\ 1 & x_{2}\\ 1 & x_{3}\\ \vdots & \vdots \\ 1 & x_{m} \end{array}\right),\;C=\left(\begin{array}{c} a\\ m \end{array}\right)$$

In Equation (11), the parameters *a* and *m* are linear with respect to the linear least square (LLS) problem. Thus, we have to solve the minimisation problem:

(12) $$C^{\prime }=arg\;\underset{C}{m}in\;O\left(C\right)$$

where the objective function O(C) is:

(13) $$O\left(C\right)={\left\|f(x)-XC\right\|}^{2}$$

in Equation (13). The components of the vector f(x) are the offsets at different positions in a projection image calculated with Algorithm 5. The least square fitting problem is solved with Eigen [20]: A C++ template library for linear algebra.

### 2.5. Optimization

Backprojection is the most time consuming part of the CT reconstruction. Therefore, we gain the most speed-up if we focus the optimization there. Because the backprojection can be executed for all voxels independently, the process can easily be parallelized. In the past, multithreaded programs running on multiple CPUs were used to achieve high performance. For more than 10 years, programmable GPUs have been available which perfectly fit with their parallel pipeline processor architecture the needs for parallel programming. With them, it is possible to perform high-performance computing on a personal computer at low costs.

When implementing the backprojection on a GPU using NVIDIA’s CUDA programming extensions for C/C++ we are faced with three main performance bottlenecks having significant potential for optimization. These are: memory access, arithmetic operation and transfer of data from CPU to GPU memory and back. In the following, we will take a closer look at optimization strategies mitigating these bottlenecks.

Memory access: GPU’s memory model knows different types of memory. The biggest one, the global memory, allows access to the complete GPU memory from all threads of a kernel program (program running on the GPU) but has the highest latency. Only global memory provides sufficient memory to upload a stack of projection to GPU memory. Textures are special GPU memory types which are bound to the global memory. Texture memory is constant and only read access is allowed. However, because of the fixed address bounding, it enables L1 caching of memory, which itself boosts global memory bandwidth. The cache usage for textures can be enhanced if we have nearby spatial locality memory access by parallel executed GPU threads [21]. In our CT reconstruction, we achieve this by backprojecting a sub-region of voxels in a slice $S_{i}$ (see Figure 5) in a block of parallel thread. The projections into the detector plane of them laying all nearby and therefore profit from L1 caching effect. Additionally, we utilize CUDA’s high speed intrinsic bi-linear on-chip interpolation of 2D and 3D textures in our implementation.

Register memory is the fastest memory on GPU. The correct utilization of register variables is a further option to optimize the backprojection speed. Register memory is dedicated to single threads and is the scarcest memory resource on GPU. An over-usage will slow down the speed of application.

Arithmetic operations: The CUDA toolkit provides highly optimized mathematical functions. Because not all of those functions fulfill the IEEE precision standard, tests should be performed to validate the results. For the usage of intrinsic functions in our implementation, see source code Listing A1.

Memory transfer: We are using asynchronous memory copy (AMC) from CPU to GPU and vice versa in the backprojection to speed up the memory transfer. CUDA provides a Asynchronous Memory Copy API for this purpose. In conjunction with CUDA streams, memory copying from host to device and vice versa together with kernel execution can run overlapped in different streams regarding the host. The dependencies on kernel execution and memory copy within one stream will still remain. The kernel has to wait for data download and upload [22]. The usage of AMC requires the allocation of non-pageable resident memory on the CPU (CUDA function: cudaMallocHost()) and an additional non-default stream. Currently, the API does not support uploading of data to texture memory. However, we can use it for the transfer of the voxel data from CPU memory to GPU memory and back (see source code Listing A4).

## 3. Results and Benchmarks

Based on the results of the benchmarks, we will reveal the parameter settings dependencies regarding backprojection performance. The benchmarks were performed on a stack of images with 2048 pixel width, 2048 pixel height and 1800 projections with the following geometry parameters: SCD of 188.0 mm, SDD of 1017.34 mm and a detector pixel size of 0.2 mm. The resulting volume has the size 2048 × 2048 × 2048 voxels. The configurations and results of the benchmarks are listed in Table 1 and Table 2. We perform the benchmarks on two computing systems:

1. NVIDIA Quadro P4000 8 GB; Processor Intel(R) Xeon(R) W-2133 CPU 3.60 GHz, 3600 MHz, 6 cores, 128 GB;
2. NVIDIA Quadro RTX 8000 48 GB; Processor Intel(R) Xeon(R) Gold 6242 CPU 2.80 GHz, 2793 MHz, 16 Cores, 768 GB.

MAP defines the number of voxel slices processed in one backprojection kernel call. The upper limit of used register variables can be controlled with the compiler parameter “Max Used Register” RN. MAP does not only controls how many slice are copied for a kernel call, it is also used as unrolling parameter in the backprojection kernel (source Listing A3). Because unrolling unwinds the loop body, it also increases register variables usage. Therefore, MAP, together with RN, must carefully be tuned. This is because when the register memory resource is becoming exhausted, the register variables will automatically be converted (spilled out) to local variables. Local variables are orders of magnitude slower than register variables. This will lead to a performance decrease. Furthermore, with MAP we can control the number of kernel calls and subsequently the total number of memory copies.

There is a maximum upper limit on non-pageable memory allocation, which cannot be controlled by the application software using Nvidia’s Cuda toolkit SDK function cudaHostAlloc() and is on a Windows-based system per default roughly 25% of the CPU memory. The default maximum limit can be changed by operating-system-specific functions. This should be done carefully, not to compromise functionality of the system, especially on multi-user systems. With the default limit, we have to split for system 1 the CPU memory stack into two parts. Therefore, the projections have to be copied twice to texture memory. The projection stack itself must be split into five batches for the backprojection and the kernel is called five times more than for system 2. System 2 needs no split at all, neither for the CPU nor the GPU memory. Thus, the performance improvement potential of AMC is higher for system 1 than for those for system 2. For system 1, with AMC alone it is ≈ 38%. Together with register usage tuning, it is ≈ 48%. For system 2, with AMC alone it is ≈ 25%. With both, it is ≈ 32%. The timing results of benchmarks B4 and B10 are showing some anomalies for system 2, which are reproducible, but the exact reason is unclear.

In summary:

We studied the performance dependency of the backprojection for the parameters with the highest impact. We can conclude that the results of a parameter optimization retrieved on specific computer system cannot directly transferred to another with a different hardware configuration. Nonetheless, we can identify trends for optimal parameter configuration independent of the hardware: Enable AMC and reduce memory copy to/from GPU by uploading (downloading) multiple voxel slices. Maximize register variable usage by loop unrolling but avoid over-usage of registers. By applying the benchmarks to both available computer systems, we found optimization parameters working on both systems as a compromise quite well with AMC copy in batches of four slices to GPU memory, two streams and RN = 36.

## 4. Discussion

In this article, we presented a tool chain which covers all necessary steps for cone-beam CT reconstruction from raw projection images. Furthermore, methods for artifact reduction and geometric error correction were introduced. The work is focused on the analytical filtered backprojection, which is a fast and reliable method for CT scans without severe artifacts for a sufficient amount of projections. Although iterative reconstruction solvers enable new opportunities to resolve problems which are hard to solve with analytical methods, the latter are still the most frequently used method in every day practice, especially if very large datasets are involved. Examples of where iterative methods are beneficial include heavy starvation artifacts, reconstruction techniques using additional models (CAD, physical, noise, …), reconstruction from real time CT with few projections and non-uniform distributed projections. However, the main disadvantages of iterative reconstructions algorithms are low speed, slow or not guaranteed convergence and the complexity for parallelization of big datasets on commonly used computers with limited GPU resources (none High Performance Cluster HPC).

Our analytical CT reconstruction tool chain is very efficient and can be applied to arbitrarily large sized projection datasets. We have extensively benchmarked the tool and have shown generic trends for optimal hardware parameter configuration to boost the performance. Although not discussed explicitly, an implementation of the backprojection for a multi CPU and GPU system is straightforward. Algorithm 3 can be used as a basis.

As we have demonstrated, parameter optimization is an expensive task and hardware dependent. Therefore implementing auto-tuning of the GPU application parameters would be beneficial for a more simple adaption to different hardware systems. Examples for GPU auto-tuning can be found in [23,24]. Optimization by profiling is a further topic not discussed yet but was performed. It is summarized in Appendix A.

## Figures and Tables

**Table 1:** Benchmarks types. Asynchronous memory copy is enabled when two streams are activated.

Benchmark	# of Streams	# Slices Copied to Kernel	Max Used Register
B1	default	1	31
B2	default	1	63
B3	default	4	31
B4	default	4	63
B5	default	8	31
B6	default	8	63
B7	2	1	31
B8	2	1	63
B9	2	4	31
B10	2	4	63
B11	2	8	31
B12	2	4	63
B13	2	4	36

**Table 2:** Processor time of backprojection for different benchmarks. Averaged over four measurements.

Graphics Card	Benchmark No.	Execution Time [s]
NVIDIA Quadro P4000	B1	264.6
NVIDIA Quadro P4000	B2	269.2
NVIDIA Quadro P4000	B3	200.4
NVIDIA Quadro P4000	B4	200.3
NVIDIA Quadro P4000	B5	186.4
NVIDIA Quadro P4000	B6	188.0
NVIDIA Quadro P4000	B7	223.5
NVIDIA Quadro P4000	B8	224.7
NVIDIA Quadro P4000	B9	148.4
NVIDIA Quadro P4000	B10	144.4
NVIDIA Quadro P4000	B11	139.5
NVIDIA Quadro P4000	B12	140.2
NVIDIA Quadro P4000	B13	140.1
NVIDIA Quadro RTX8000	B1	101.6
NVIDIA Quadro RTX8000	B2	97.2
NVIDIA Quadro RTX8000	B3	86.3
NVIDIA Quadro RTX8000	B4	125.4
NVIDIA Quadro RTX8000	B5	98.1
NVIDIA Quadro RTX8000	B6	89.0
NVIDIA Quadro RTX8000	B7	91.3
NVIDIA Quadro RTX8000	B8	87.3
NVIDIA Quadro RTX8000	B9	73.9
NVIDIA Quadro RTX8000	B10	115.4
NVIDIA Quadro RTX8000	B11	86.8
NVIDIA Quadro RTX8000	B12	76.5
NVIDIA Quadro RTX8000	B13	68.2

**Figure A1:**
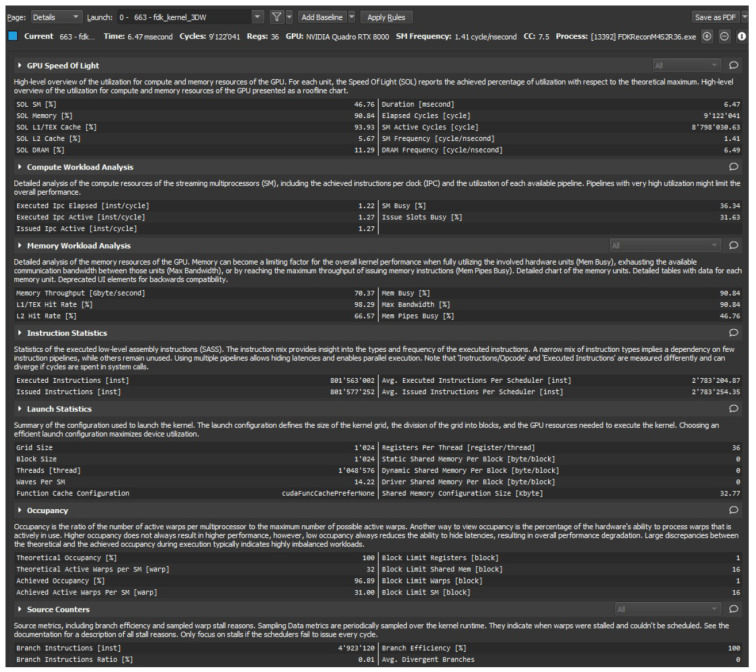
Backprojection kernel profiling using Nsight Compute on Nvidia Quadro RTX 8000.

**Listing A1:**
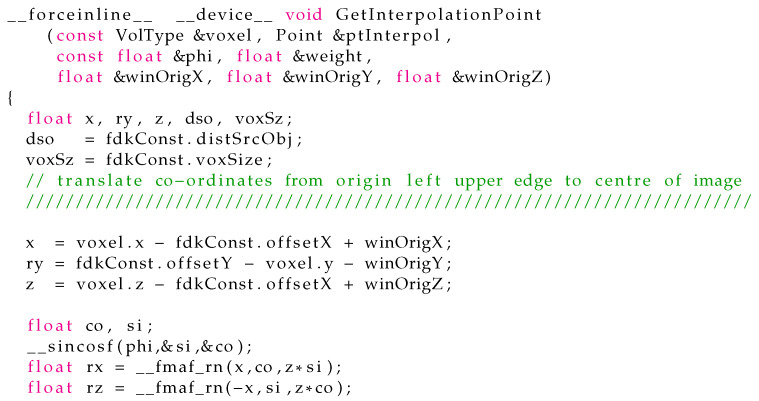

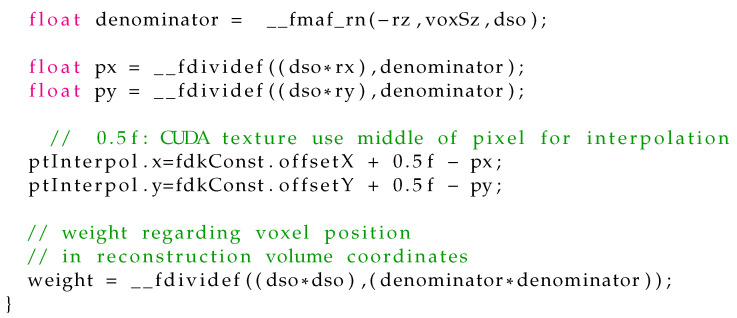
Function to retrieve intersection point in projection and determine voxel weight.

**Listing A2:**
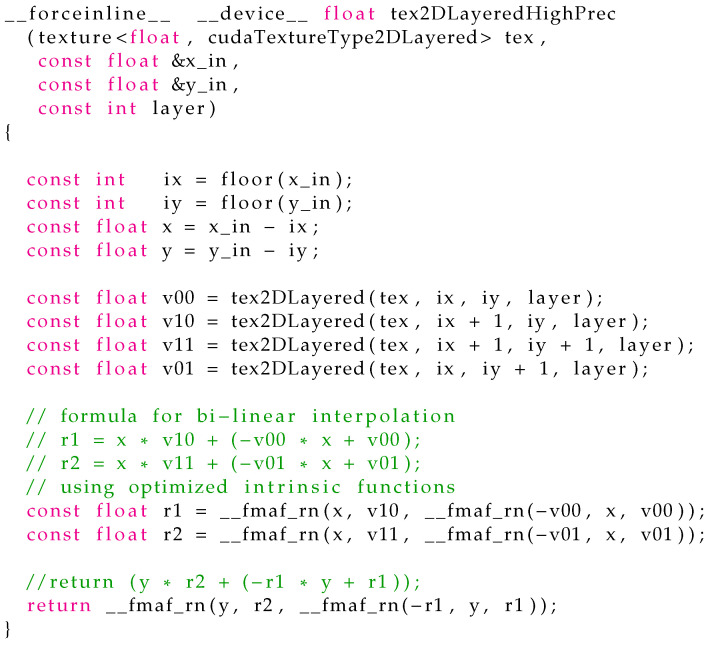
Bi-linear interpolation with 32 bit float precision; the used fast intrinsic __fmaf_rn() is IEEE compliant.

**Listing A3:**
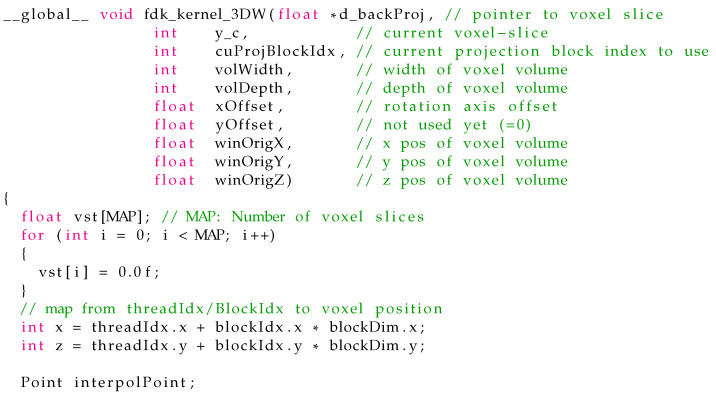

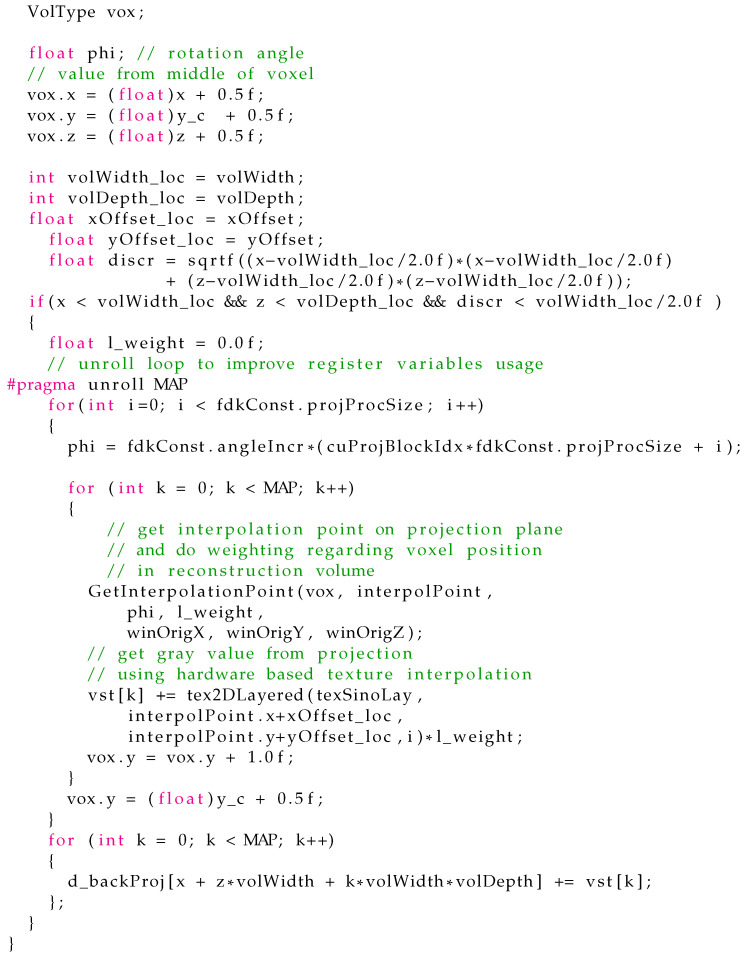
Source code for default backprojection kernel function.

**Listing A4:**
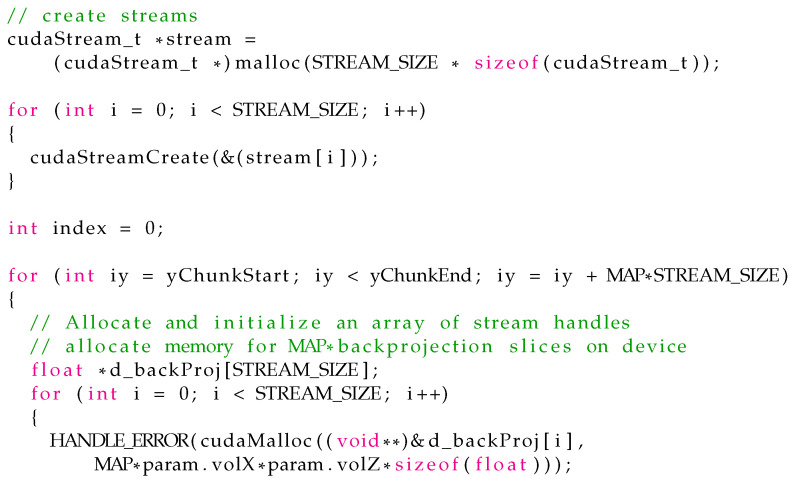

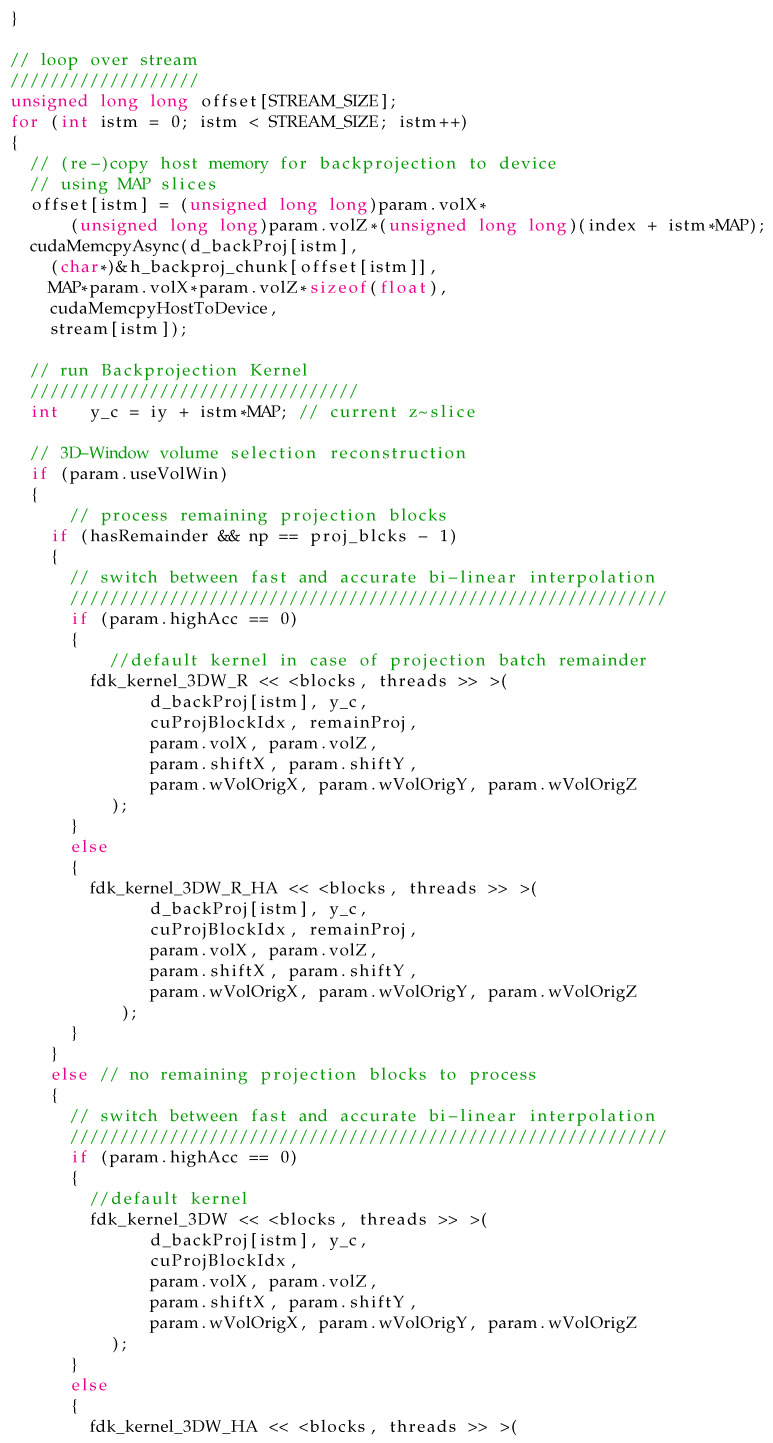

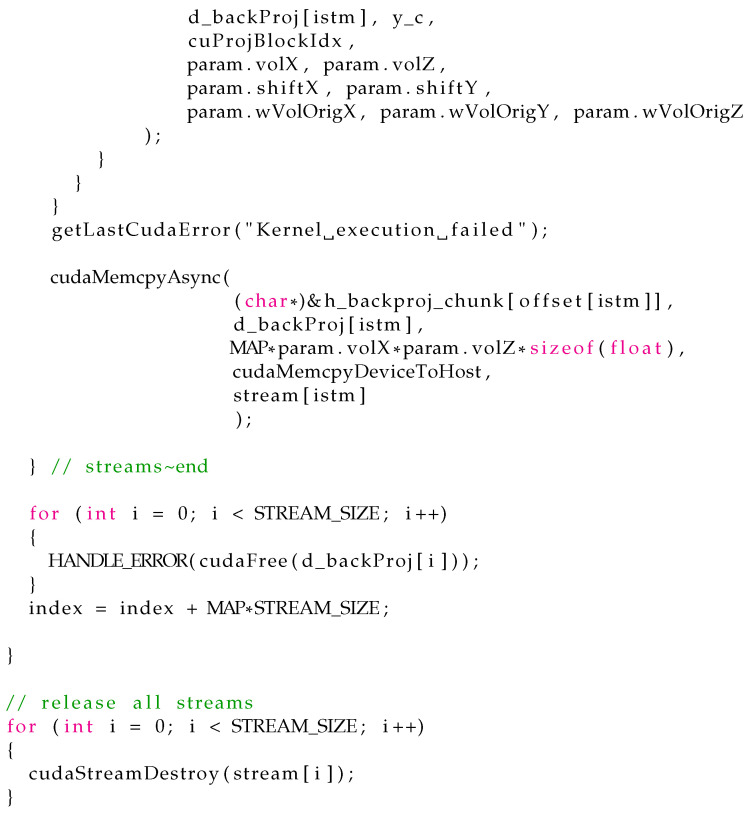
Code snippet: Asynchronous memory copy handling and kernel calls.

## Data Availability

Not applicable.

## Abbreviations

The following abbreviations are used in this manuscript:

AMC	Asynchronous memory copy
BHC	Beam hardening correction
CT	Computed Tomography
DLR	Detector line ratios
FDK	Feldkamp, Davis and Kress reconstruction algorithm
GPU	Graphics processing unit
MF	Median Filter
ROI	Region of interest
SCD	Source rotation center distance
SCM	sensitivity correction matrix
SDD	Source detector distance SDD

## Appendix A. Profiling Results

Figure A1 shows the results of profiling the backprojection GPU code using the application NVIDIA Nsight Compute Version: 2021.1.1.0 on the computer system 1 (see Section 3). To enable the profiling of benchmark B13, we had to down-sample the input data by a factor of two and reduce the reconstructed volume size to 1024 × 1024 × 1024 voxels due to the computing overhead of the profiling tool Nsight Compute.

In the following, we will give a brief interpretation of the result.

As discussed in Section 2.5, crucial for fast texture memory access is the reuse of the L1 cache. The result for “L1/tex Cache compute utilization” of 94% and a “texture hit rate” of 98% shows that we achieved a high reuse rate. CUDA’s programming model defines a thread hierarchy (see details in [25]). A grid contains thread blocks which are hosts for the threads. These parameters are used for the kernel launch. We configured the parameters that the kernel runs with the maximal number of 1024 threads per block. This leads to an Occupancy value of 96%, which indicates a high computing workload of the GPU processor.

## Appendix B. Source Code Listing

The essential source code of the backprojection is presented in the following. It is extracted from the original code and is written in the CUDA specific C++-language.

In Listing A3, we present the code of the default backprojection kernel function fdk_kernel_3DW() using Nvidias tex2DLayered() function for texture fetching and interpolation. For the other kernel functions, please visit the source code. MAP is the number voxel slices, which is loaded for one kernel call and is used as an unrolling parameter.
